# Automatic Recognition of Element Classes and Boundaries in the Birdsong with Variable Sequences

**DOI:** 10.1371/journal.pone.0159188

**Published:** 2016-07-21

**Authors:** Takuya Koumura, Kazuo Okanoya

**Affiliations:** 1 Department of Life Sciences, Graduate School of Arts and Sciences, The University of Tokyo, Tokyo, Japan; 2 Research Fellow of Japan Society for the Promotion of Science, Tokyo, Japan; 3 Cognition and Behavior Joint Laboratory, RIKEN Brain Science Institute, Saitama, Japan; Rutgers University, UNITED STATES

## Abstract

Researches on sequential vocalization often require analysis of vocalizations in long continuous sounds. In such studies as developmental ones or studies across generations in which days or months of vocalizations must be analyzed, methods for automatic recognition would be strongly desired. Although methods for automatic speech recognition for application purposes have been intensively studied, blindly applying them for biological purposes may not be an optimal solution. This is because, unlike human speech recognition, analysis of sequential vocalizations often requires accurate extraction of timing information. In the present study we propose automated systems suitable for recognizing birdsong, one of the most intensively investigated sequential vocalizations, focusing on the three properties of the birdsong. First, a song is a sequence of vocal elements, called notes, which can be grouped into categories. Second, temporal structure of birdsong is precisely controlled, meaning that temporal information is important in song analysis. Finally, notes are produced according to certain probabilistic rules, which may facilitate the accurate song recognition. We divided the procedure of song recognition into three sub-steps: local classification, boundary detection, and global sequencing, each of which corresponds to each of the three properties of birdsong. We compared the performances of several different ways to arrange these three steps. As results, we demonstrated a hybrid model of a deep convolutional neural network and a hidden Markov model was effective. We propose suitable arrangements of methods according to whether accurate boundary detection is needed. Also we designed the new measure to jointly evaluate the accuracy of note classification and boundary detection. Our methods should be applicable, with small modification and tuning, to the songs in other species that hold the three properties of the sequential vocalization.

## Introduction

Sequential vocalizations, in which voices are produced sequentially, have been a target of wide variety of researches. This is not only because they include human spoken language, but also because they serve as excellent models for precise motor control, learning, and auditory perception.

Birdsong is one of the most complex and precisely controlled sequential vocalizations, and has been widely and intensively studied [1–3]. Birdsong, as well as most of other sequential vocalizations, has several distinct properties. First, usually a song is a sequence of discrete vocal elements (called notes) [4]. Thus, by grouping similar notes into a single class, it is possible to convert songs into symbol sequences of note classes. Notes in a single class are considered to be generated by the same set of commands in motor neurons, which leads to the similar patterns of muscle activation to the similar acoustic outputs [5–8]. It is also known that auditory stimuli of notes in the same class invoke similar activation patterns in the auditory brain areas [9]. Second, temporal structure of the song is precisely controlled. Neural activities that are time-locked to a particular timing in a particular note class have been found during song production and perception [10–13]. Other studies have shown that temporal patterns of birdsong are constructed upon multiple levels of variability from local to global ones [14–16]. Thus, in analyzing birdsong it is important to accurately extract timing information such as note onsets and offsets. Finally, notes are sequenced not randomly but according to a certain probabilistic rule. Usually rules for note sequencing are unique to individuals and acquired by learning [17–21]. This rule for note sequence production is called song syntax. Taken together, in analyzing birdsong it is important to group notes into classes, extract timing information, and consider song syntax.

In behavioral and physiological studies on animal vocalization it is not a rare case when vocalizations in several days (or months) are to be analyzed [22,23]. For example a Bengalese finch, one of the model species for sequential vocalization, typically sings for ten minutes to one hour totally in a day, consisting of 5–30 thousand notes (depending on individuals), resulting in tens of hours of songs including hundreds of thousand notes in several days to be analyzed. Therefore for efficient analysis of vocalizations an accurate and robust automatic recognizer is strongly desired.

As stated above, in many cases of studies on sequential vocalizations such as birdsong, it is important to extract temporal information as well as its contents. This is one big difference and difficulty in recognizing sequential vocalizations for biological research compared to ordinary human speech recognition for application purposes, in which usually the priority is to convert sound data into text sequences and thus word or phoneme boundaries are not very much important [24,25]. On the contrary, vocalizations in non-human animals usually consist of smaller number of element classes and their combination patterns compared to human spoken language, which makes recognition easier in terms of pattern complexity. Therefore developing automatic recognizers of sequential vocalizations specialized for biological purposes, not just blindly using the methods for human speech recognition, is important for further research on animal vocalization.

Several previous studies have performed automatic recognition of birdsong, using dynamic time warping (DTW) [26–28], a hidden Markov model (HMM) [27], or a support vector machine (SVM) [29]. In machine learning in general, it is crucial to construct good representations of the data that separate data classes well in the feature space. In the previous studies, sound spectra [26–28], mel-frequency cepstral coefficients (MFCC) [27], a small set of acoustic features [30], and a large number of features including spectra, cepstra, and their changes [29] have been used to describe properties of songs. However, there is no good reason to use MFCC in birdsong recognition because it has been designed for human speech. Also, it is not known whether the specific features used in the previous studies are suitable for vocalizations of other species as well. Although a SVM can automatically select good features from a large set of features, the problem of considering the initial feature set still remains. The desirable methods are ones that can automatically extract good features from data without manually engineering a set of features.

In the present study, to fulfill the three requirements stated above, we employed a hybrid model of a multi-layered neural network and an HMM, with which high performances have been achieved in human speech recognition [25]. A multi-layered neural network is known to have a capacity to find good representations of data by machine learning [31], making it possible to achieve robust note classification. Specifically we used deep convolutional neural network (DCNN) to handle long continuous sounds [32–34]. An HMM is good at handling variable sequences produced according to probabilistic syntax rules. Note boundaries were detected either with an HMM or by thresholding of amplitude and duration of note and silent intervals. Performances of the recognizers were evaluated by cross-validation with three types of error rates designed to capture accuracy of note classification, boundary detection, and both of them.

## Results

### Data sets

In total songs in 13 birds were recorded. All notes in recorded songs were manually located and classified with the help of machine learning techniques (see materials and methods). Songs in two birds which had more than 1% of manually unrecognizable notes were discarded. The fractions of unrecognizable notes in the discarded birds were 1.44% and 1.52%. Songs in the remaining 11 birds were used for the following evaluation. The average ± standard deviation of the total song duration in 11 birds was 40.7 ± 18.4 minutes. The number of total notes and the number of note classes was 17930.9 ± 8941.8 and 8.1 ± 3.9, respectively. Songs in each bird were individually processed because songs were largely different among birds.

The entire data sets were randomly divided into three for three-fold cross-validation. In machine learning in general, the larger the training data set the more generalization ability is obtained. It has been also the case in the previous study on birdsong recognition [29]. In the current study we compared the recognition results trained on two and eight minutes of training data randomly selected from the non-validation data set (2 / 3 of the whole data). The number of notes in two and eight minutes of training data was 862.5 ± 60.8 and 3474.2 ± 241.6, respectively.

### Three steps in birdsong recognition

**Fig 1:**
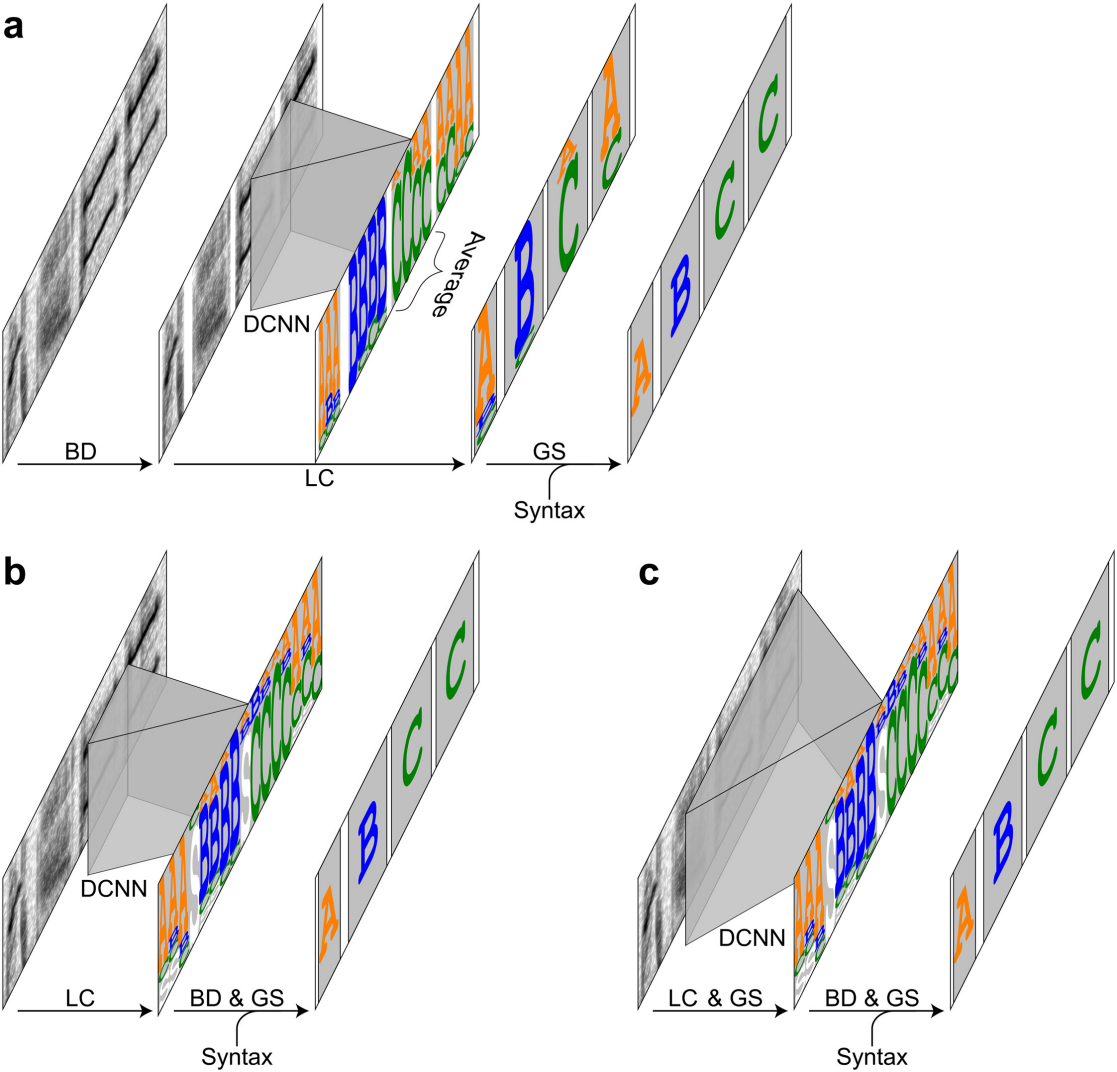
Three arrangements of methods for birdsong recognition. Flow diagrams for the three arrangements compared in this study. (a) BD → LC → GS arrangement. The colored letters A, B, and C indicate the note classes, and the white regions indicate the detected inter-note silent intervals. (b) LC → BD & GS arrangement. The white letter S indicates the silent intervals. (c) LC & GS → BD & GS arrangement.

In this study we divided the automatic recognition of birdsong into three sub-problems, each of which corresponds to one of the three properties of birdsong stated in the introduction section. First, notes must be correctly located in the continuous sound recordings by detecting note boundaries (note onsets and offsets). We call this step “boundary detection”. Second, each note must be classified into a given number of classes (or the class for silence). We call this step “local classification”. The combination of boundary detection and local classification is equivalent to object spotting or semantic segmentation in two-dimensional object recognition [35,36]. Finally, outputs of the local classification are sequenced according to given song syntax. We call this step “global sequencing”. In the global sequencing step misclassifications of the local classifier were corrected by top-down information of the song syntax. The local classification and the global sequencing step can be seen as a bottom-up path and a top-down path in the song recognition, respectively. In the current study, to efficiently perform global sequencing, what was done in the local classification step was actually soft classification. In other words, scores that represented the probability of the note classes were assigned to the local inputs. Then, the scores obtained in the local classification step were used in the following global sequencing step to determine output note sequences (Fig 1).

To accomplish the song recognition, several ways of combining or arranging these steps are possible. In this study, we compared three different arrangements (Fig 1). In the first arrangement, boundary detection, local classification, and global sequencing were sequentially conducted (Fig 1a). In the second arrangement, local classification was conducted before simultaneously conducting boundary detection and global sequencing (Fig 1b). In the last arrangement, local classification and global sequencing were simultaneously conducted before the simultaneous boundary detection and (another) global sequencing (Fig 1c). The last arrangement included global sequencing twice by different algorithms. Hereafter we call these three arrangements “boundary detection → local classification → global sequencing (BD → LC → GS)”, “local classification → boundary detection & global sequencing (LC → BD & GS)”, and “local classification & global sequencing → boundary detection & global sequencing (LC & GS → BD & GS)”. Note that different ordering of the three steps does not mean same specific algorithms can be used for the same steps in the different arrangements. For example, the number of output classes in local classification was approximately three times larger in the LC → BD & GS and LC & GS → BD & GS arrangements than in BD → LC → GS arrangements because notes should be divided into three parts for accurate boundary detection performed at the same time of global sequencing.

### Evaluation

Accuracy of the recognition was evaluated using the following three measures. To evaluate the accuracy of note classification, the Levenshtein distance between the output label sequence and the corresponding true label sequence was computed. The Levenshtein distance is the minimum number of operations of insertion, deletion, and replacement that are needed to convert one sequence into another. The actual measure used for the evaluation was the total Levenshtein distance divided by the total number of notes in the true sequences. This measure is equivalent with word ER in human speech recognition. Thus we call this measure note ER in this paper.

**Fig 2:**
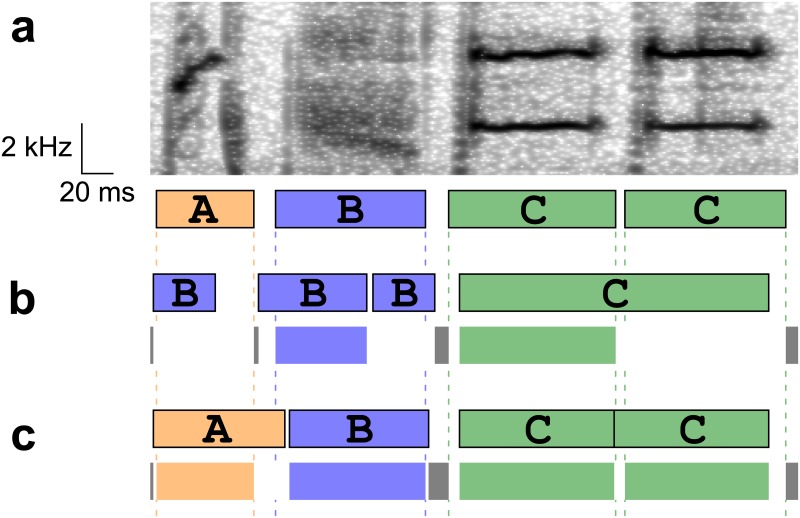
Note & timing error rate. An example of an input, a true sequence, output sequences, and correctly recognized intervals that would be obtained in computing note & timing error rate. (a) An example spectrogram and the true labels and boundaries. Note classes are indicated by letters. (b) An example recognition output (upper), and the correctly recognized intervals (lower). In the correctly recognized intervals, colored bars indicate the correctly recognized note intervals, longest overlaps with the true label intervals. Gray bars indicate the correctly recognized silent intervals. The correctly recognized intervals appear to capture the performances properly even in such cases that a single note is recognized as two (notes B) and two notes are recognized as one (notes C). In both cases either of two overlapping intervals (the one with a longer overlap) was counted as correctly recognized intervals. There could be a case in which no matched interval is assigned (note A). (c) Another example showing the recognition outputs and the correctly recognized intervals with lower note & timing error rate.

The note ER is designed to measure the difference of two symbol sequences, but does not have a capacity to capture the difference of note boundaries. The straightforward measure for jointly evaluating the accuracy of note classes and boundaries might be the total length of the time points in which the recognizer assigned different classes from those in the true sequences. However, such a measure cannot penalize the case in which two successive notes with identical classes were incorrectly recognized as one long note. Another measure might be the sum of the distances from the note onsets in the output sequences to the onsets of the nearest notes with the same classes in the true sequences plus the distances between the offsets. This measure fails if some note classes in one sequence do not exist in another. In this study, we devised the new measure for jointly evaluating the note classes and boundaries without any specific constraints (Fig 2). First, for each true note interval, an output note with the same class and with the longest overlap with the true one, if any, was matched. The overlapped sections in the matched intervals were regarded as the correctly recognized note intervals. Next, for each silent interval in the true sequence, the correctly recognized silent intervals were defined as the sections in which no classes were assigned in recognition. Finally, the total length of the correctly recognized note and silent intervals was divided by the total length of the input sequences, and subtracted from one. Hereafter we call this measure as the note & timing ER because this measure captures both classification and timing errors.

$$\text{Note}\&\text{timing ER }=1-\frac{\text{total length of the correctly recognized intervals}}{\text{total length of the input sequences}}$$

Finally, to evaluate the accuracy of timing recognition, note & timing ER was computed for the true and output sequences ignoring note classes, focusing only on the timing information. In this paper this measure is called timing ER.

Although these three error rates, note ER, timing ER, and note & timing ER, are well-defined and sound measures to capture the corresponding accuracies, these errors are not very much straightforward or intuitive. Thus, we also computed other measures called identification ER and duration ER (S1 Fig, S1 Table). These measures are based on the notes in true and output sequences that are nearest to each other. Thus, although sometimes the nearest notes cannot be defined, these measures are straightforward and easy to understand.

### Song recognition in the BD → LC → GS arrangement

**Fig 3:**
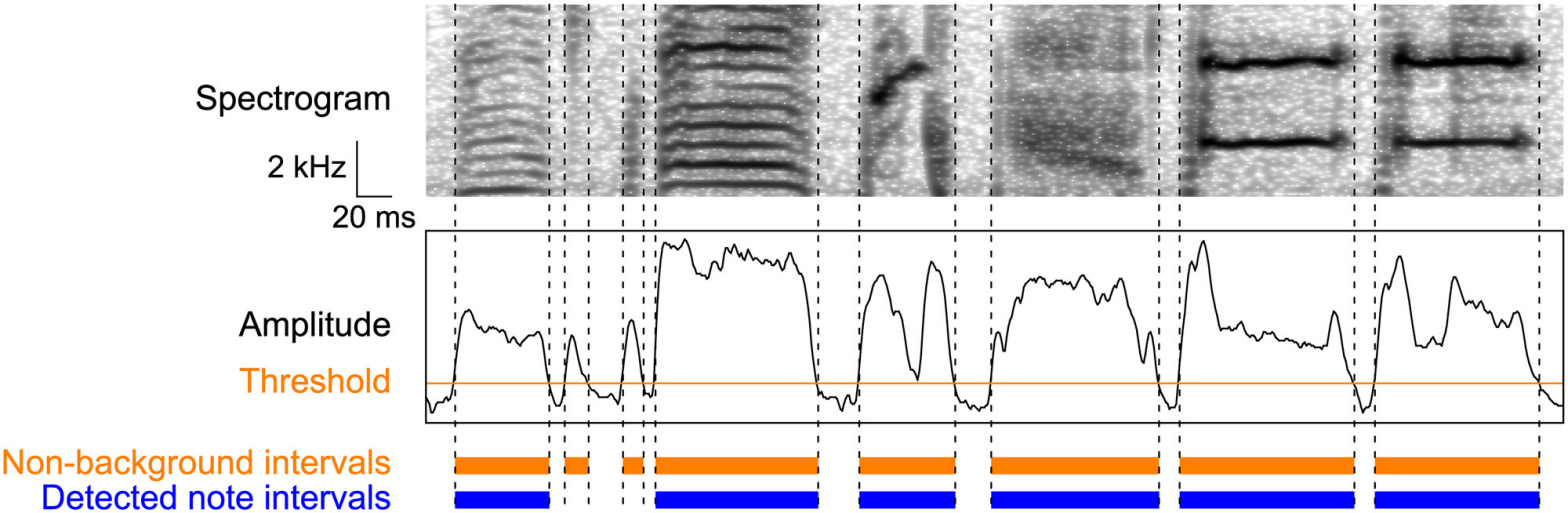
Boundary detection by thresholding. In the BD → LC → GS arrangement, note boundaries were detected using sound amplitude and interval duration. From top to bottom: input spectrogram, amplitude envelope (black line) and threshold (orange line), non-background intervals (orange bars), detected note intervals (blue bars).

**Fig 4:**
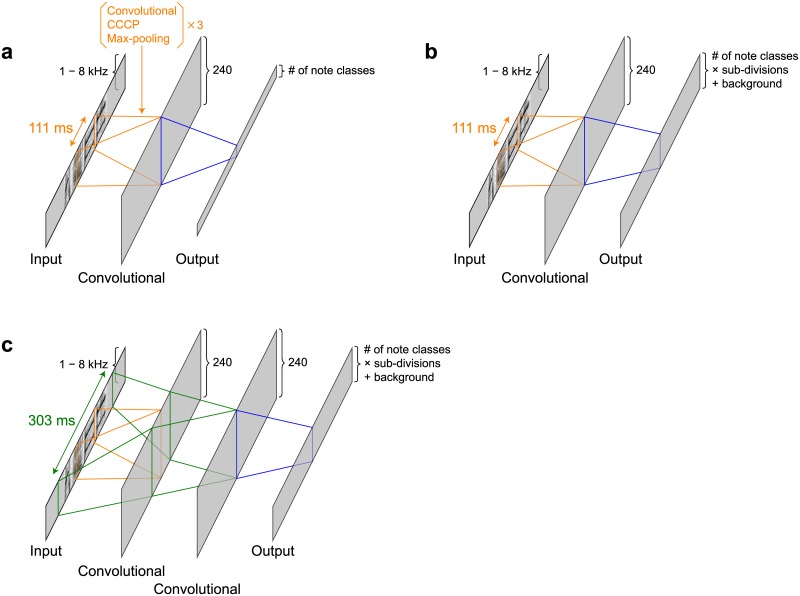
Local classification with a deep convolutional neural network. Local classification was conducted with a deep convolutional neural network. Input, intermediate, and output layers were shown in gray rectangles. The range of the input used for computing a single time point in the following layer is shown in colored lines. (a) BD → LC → GS arrangement. (b) LC → BD & GS arrangement. (c) LC & GS → BD & GS arrangement.

**Fig 5:**
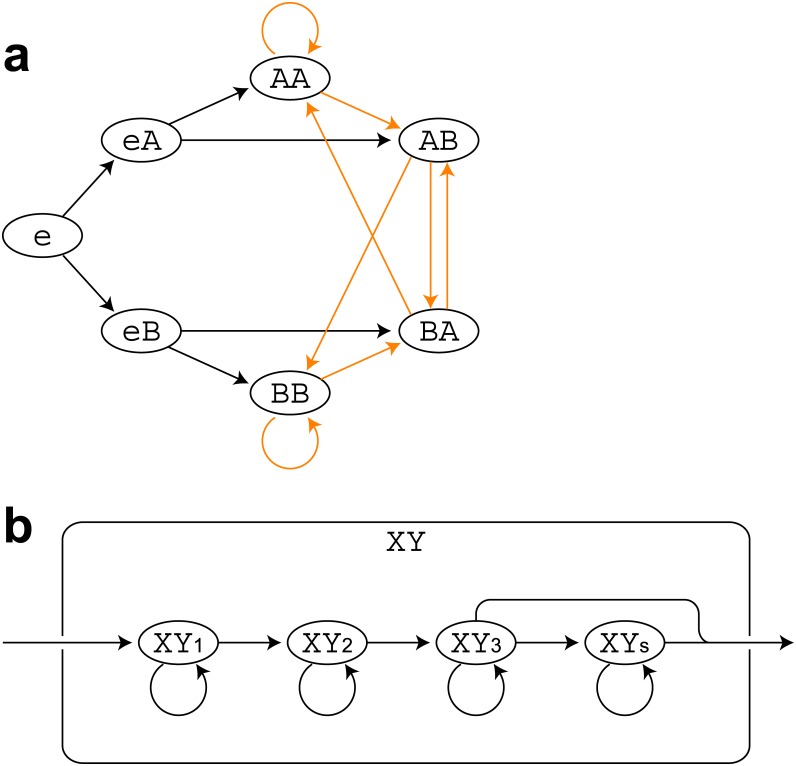
Syntax models used in HMMs. Schematic diagrams of song syntax modeled with a second-order Markov model. In this figure examples with two note classes (A & B) are shown. (a) A transition diagram in the BD → LC → GS arrangement. The initial state is indicated by the letter “e”. The transition probabilities of orange arrows were computed from the training data sets. Those of black arrows were uniformly distributed (ie. all transition probabilities from states “e”, “A”, and “B” are 0.5). Sequence generation is allowed to stop at any states. (b) In the LC → BD & GS and the LC & GS → BD & GS arrangements, each state in (a) except the initial state was divided into four. The letter “X” and “Y” denote any note classes or the initial state.

In the BD → LC → GS arrangement (Fig 1a), first, note boundaries were located as the intervals with amplitude and duration larger than certain thresholds (BD step; Fig 3). Then sound spectrograms were fed into a DCNN to obtain scores for a given number of note classes at every time point (LC step; Fig 4a). The scores assigned by the DCNN to a time point represented the probability of the class labels assigned to the local input with the length of 111 ms centered on the time point. The scores were averaged in each detected note interval. Finally, the averaged likelihoods and the pre-computed song syntax (Fig 5) were fed into an HMM to determine the output classes for the note intervals that maximize the total likelihood throughout the sequences (GS step).

**Fig 6:**
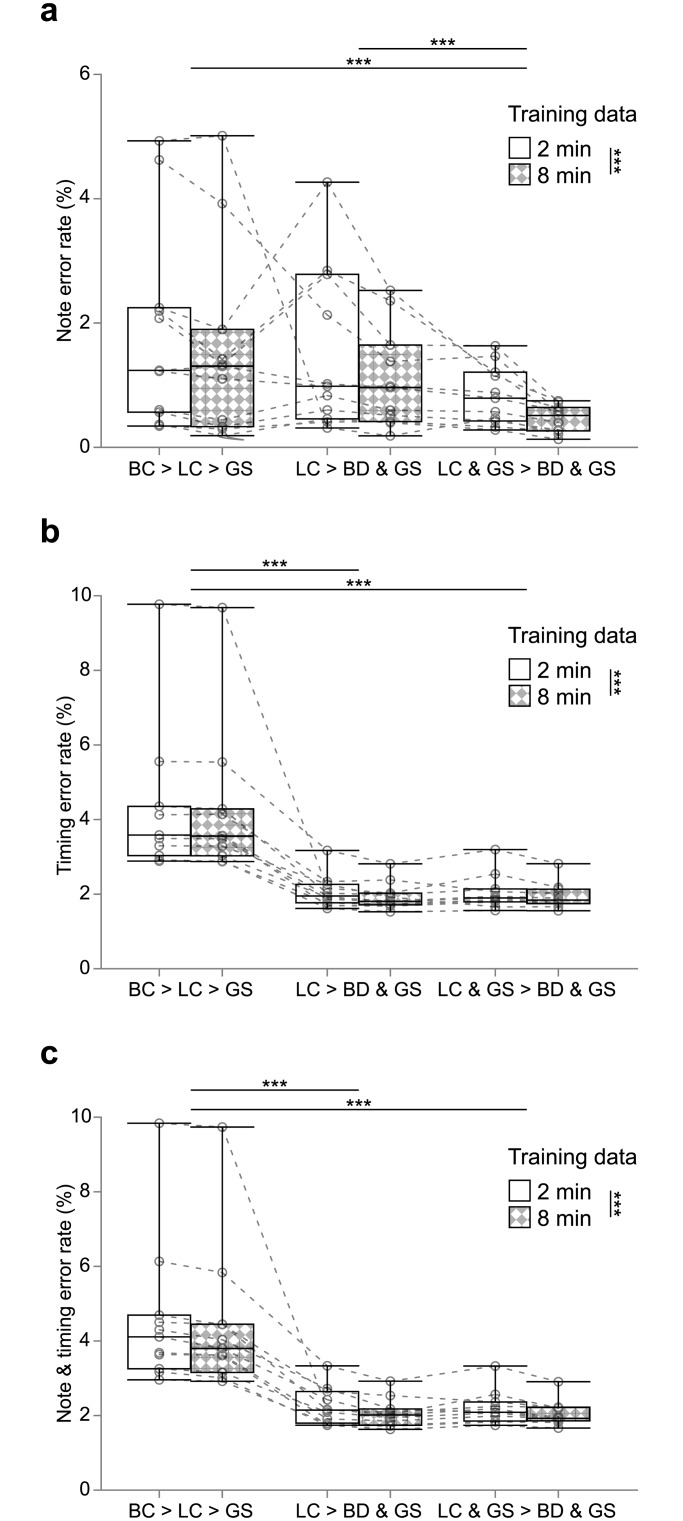
Validation errors. (a) Note ERs of the results trained on two and eight minutes of training data sets. ERs in each bird are shown in open circles. (b) Timing ERs. (c) Note & timing ERs. ***: *p* < 0.001.

**Fig 7:**
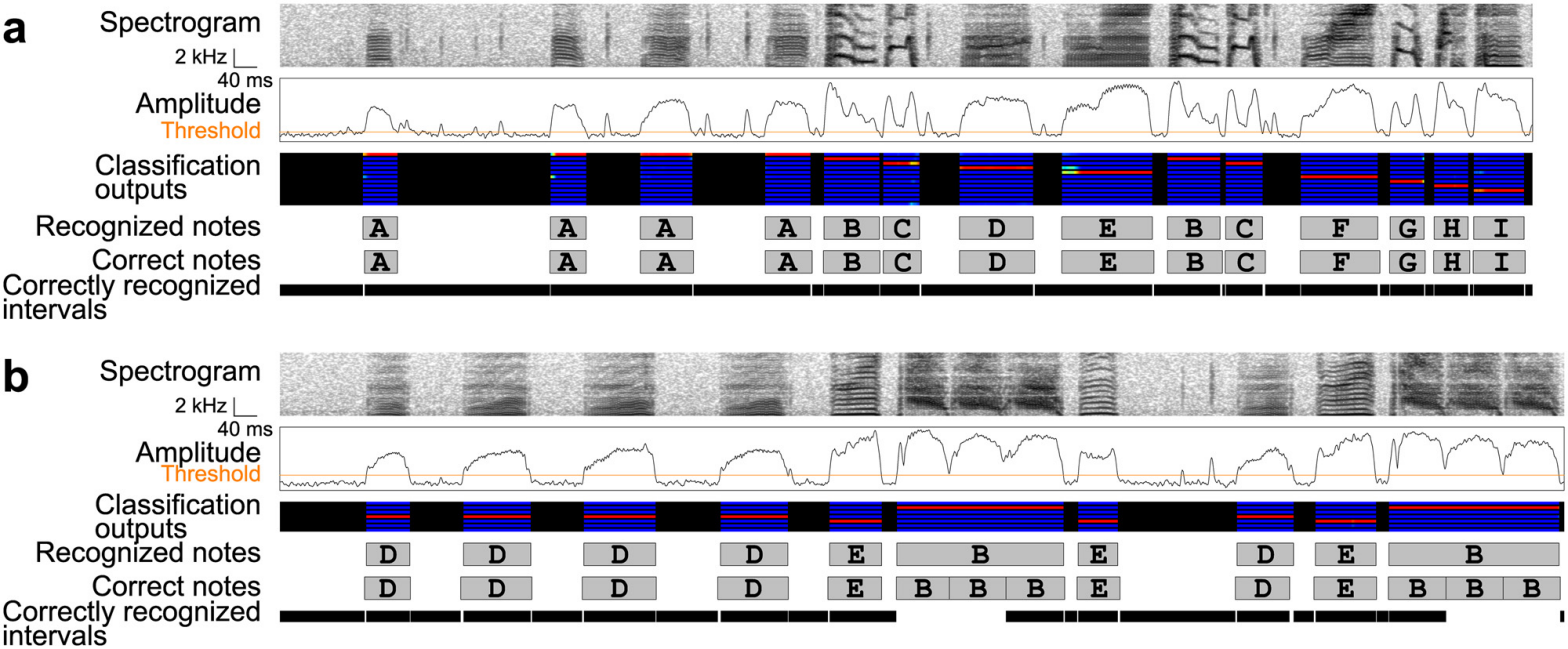
Recognition results in the BD → LC → GS arrangement. (a) A recognition result in one bird. From upper to lower: an input spectrogram, amplitude, outputs of local classification, recognized note intervals, true note intervals, and correctly recognized intervals. Rows in the classification outputs correspond to the note classes. The black areas are putative silent intervals detected in the boundary detection step. Gray rectangles with letters indicate note intervals and classes. The correctly recognized intervals are indicated by black bars. (b) A result in another bird with poorer recognition accuracy.

The average note ERs were as low as 1.86% with 2 minutes of training data and 1.57% with 8 minutes of training data (Table 1), ranging from 0.19% to 5.01% (Fig 6a), whereas the average timing ERs were 4.24% and 4.21% with 2 minutes and 8 minutes of training data, among which ERs in one bird reached nearly 10% (Fig 6b). These results suggest that note boundaries are not accurately detected by the BD → LC → GS arrangement. Indeed the recognition result in the bird with the highest note & timing ERs shows that boundaries between notes with almost no silence could not be detected by the thresholding method (Fig 7b). Obviously the note & timing ERs are lower-bounded by the errors of boundary detection, measured with the timing ERs. In contrast, if boundary detection worked well, the following classification and sequencing were successful (Fig 7a).

**Table 1:** Average note ERs, timing ERs, and note and timing ERs.

ERs (%)	Note		Timing		Note & timing	
Training data length (minutes)	2	8	2	8	2	8
BD → LC → GS	1.86	1.57	4.24	4.21	4.57	4.42
LC → BD & GS	1.51	1.09	2.07	1.94	2.25	2.06
LC & GS → BD & GS	0.84	0.46	2.04	1.95	2.21	2.06

### Song recognition in the LC → BD & GS arrangement

**Fig 8:**
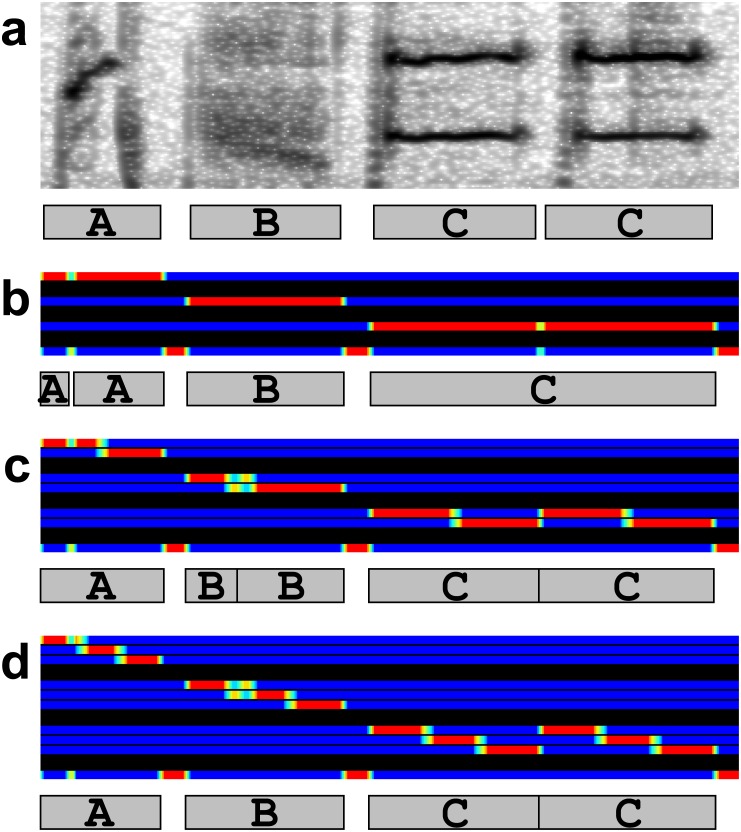
Sub-divisions in notes. (a) An example input spectrogram (upper) and the true note intervals (lower). Gray rectangles with letters indicate note intervals with note classes. (b) Example outputs of a DCNN without sub-division in notes (upper) and the recognized sequence (lower). First three rows in the DCNN outputs correspond to three note classes and the last to the class for silence. (c) Example outputs with notes divided into two parts. First six rows in the DCNN outputs correspond to three note classes with two sub-divisions. The last row corresponds to the silence. (d) Example outputs with notes divided into three parts. First nine rows in the DCNN outputs correspond to the three note classes with three sub-divisions.

In the LC → BD & GS arrangement (Fig 1b), first, sound spectrograms were fed into a DCNN to obtain scores at every time point as in the BD → LC → GS arrangement. The difference from the BD → LC → GS arrangement was the number of the output classes in the DCNN (Fig 4b). Each note was divided into three sub-classes corresponding to the beginning, middle, and end of the note because performing boundary detection based on the outputs of local classification requires these three parts to be distinguished from one another. After local classification the scores at every time point are fed into an HMM to obtain note boundaries and output classes that maximize the total likelihood of the sequences. Note that in contrast with the BD → LC → GS arrangement, the outputs of the DCNN were not averaged across time because boundary detection was not yet done at the time of the local classification. To accurately detect note boundaries with an HMM, each note was divided into three parts with the same duration (Fig 8, S3 Fig). Accordingly, the number of output classes in the DCNN was three times the number of the note classes plus one (silence). Note onsets were defined as the first time points at which first sub-divisions of the note appeared after the third sub-divisions or the silence. Note offsets were defined as the last time points of the third sub-divisions.

**Fig 9:**
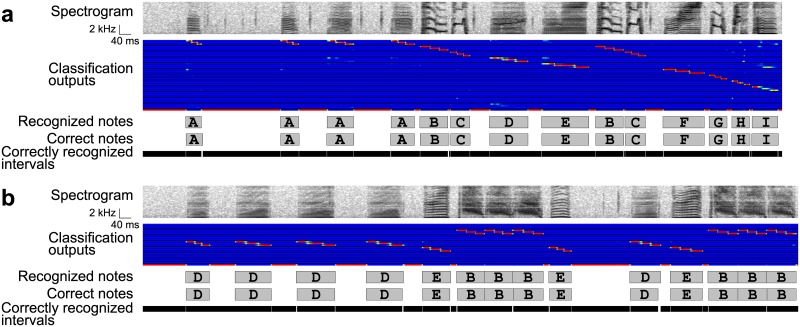
Recognition results in the LC → BD & GS arrangement. (a) A recognition result in one bird. From upper to lower: an input spectrogram, outputs of local classification, recognized note intervals, true note intervals, and correctly recognized intervals. Rows in the classification outputs correspond to twelve note classes with three sub-divisions. The bottom row indicates the class for the background noise. Gray rectangles with letters indicate note intervals and classes. The correctly recognized intervals are indicated by black bars. (b) A result in another bird.

Examples of the recognition results show that sub-divisions in notes were correctly classified by the DCNN, resulting in correct sequencing and boundary detection with the HMM (Fig 9), even in the data poorly recognized with the BD → LC → GS arrangement (Fig 9b). Timing ERs and note & timing ERs were lower than those in the BD → LC → GS arrangement (for both types of ERs, Wilcoxon signed-rank test, *W* = 253, *p* = 1.43×10^−6^, adjusted by the factor of three), suggesting that the LC → BD & GS arrangement is more suitable for recognizing precise temporal information. Note ERs were not significantly different from those in the BD → LC → GS arrangement (*W* = 130, *p* > 1, adjusted by the factor of three).

### Song recognition in the LC & GS → BD & GS arrangement

The LC & GS → BD & GS arrangement was almost same as the LC → BD & GS arrangement except for the architecture of the DCNN (Figs 1c and 4c). To include the syntax information that spanned over more than one notes in the DCNN, additional layer was inserted in the DCNN, resulting in the width of the input time window widened to 303 ms, roughly covering three successive notes.

**Fig 10:**
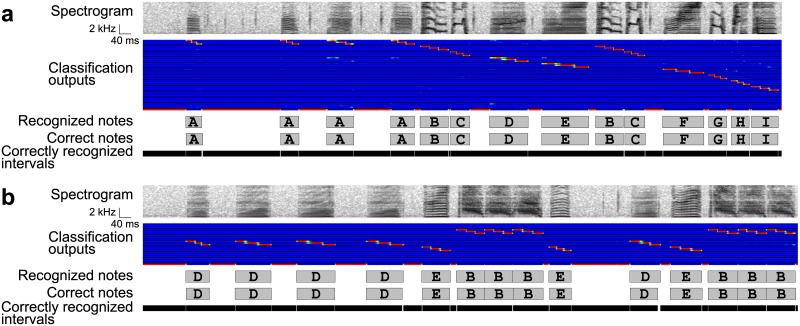
Recognition results in the LC & GS → BD & GS arrangement. (a) A recognition result in one bird. From upper to lower: an input spectrogram, outputs of local classification, recognized note intervals, true note intervals, and correctly recognized intervals. Rows in the classification outputs correspond to twelve note classes with three sub-divisions. The bottom row indicates the class for the background noise. Gray rectangles with letters indicate note intervals and classes. The correctly recognized intervals are indicated by black bars. (b) A result in another bird.

Examples of the recognition results show that recognition was accurately performed in the LC & GS → BD & GS arrangement as well as in the LC → BD & GS arrangement (Fig 10). All types of ERs were lower than those in the BD → LC → GS arrangement (for all types of ERs, Wilcoxon signed-rank test, *W* = 253, *p* = 1.43×10^−6^, adjusted by the factor of three; Fig 6), and the note ERs were lower than those in the LC → BD & GS arrangement (*W* = 247, *p* = 2.00×10^−5^, adjusted by the factor of three; Fig 6a). These results demonstrated the capacity of a DCNN to capture the syntax information ranging over multiple successive notes. Timing ERs and note & timing ERs were not significantly different from those in the LC → BD & GS arrangement (for timing ERs, *W* = 146; for note & timing ERs, *W* = 130; for both types of ERs, *p* > 1, adjusted by the factor of three).

### Summary of the validation errors

Note ERs were the lowest in the LC & GS → BD & GS arrangement. Timing ERs and note & timing ERs were the highest in the BD → LS → GS arrangement. The larger the training data, the lower both types of errors were (for note ERs, Wilcoxon signed-rank test, *W* = 544, *p* = 2.39×10^−6^; for timing ERs, *W* = 522, *p* = 1.53×10^−5^; for note & timing ERs, *W* = 560, *p* = 5.64×10^−7^; Fig 6), as has been shown in most of other machine learning studies.

## Discussion

In the current study we evaluated the three different arrangements to automatically recognize songs in Bengalese finches, and with two arrangements achieved sufficiently low validation errors (~ 2%) for practical use in biological studies (Table 1, Fig 6). All arrangements used a DCNN for local classification and an HMM for global sequencing, demonstrating the effectiveness of the hybrid use of a DCNN and an HMM for recognizing birdsong as well as human speech in the previous studies [25,37,38]. To the best of our knowledge this is the first time that a hybrid DCNN/HMM model was applied to automatic recognition of the birdsong.

The note & timing ERs were higher in the BD → LC → GS arrangement than in the other two arrangements, suggesting that boundary detection should be performed with an HMM rather than by amplitude and duration thresholding. The note ERs were also higher in the BD → LC → GS arrangement but were acceptably low for practical use (Table 1). One advantage of the BD → LC → GS arrangement is that by abandoning boundary detection with an HMM the number of target classes in the DCNN decreases approximately by a factor of three, making the computation for classification easier and faster. The faster the computation, the finer the parameters could be tuned, possibly leading to better generalization. Some readers might think that the problem would get easier with splitting notes into three because three parts of the notes are usually spectrally different and splitting notes resulted in making inputs in each category more uniform. Indeed, this may be the case for some classifiers such as SVM, linear discriminant analysis, and neural networks without hidden layers. However, uniformity of data within a single category does not affect the performances in multi-layered neural networks such as DCNN because these classifiers are highly nonlinear and able to handle nonlinear mappings from inputs to outputs. Moreover, slightly better recognition in the LC & GS → BD & GS step compared to that in LC → BD & GS step revealed the power of a DCNN in handling data with complex and hierarchical structure. Therefore we propose that either of the BD → LC → GS or the LC & GS → BD & GS arrangement should be employed according to the objectives: when accurate note classification is the first priority and the information of note boundaries were not important, the BD → LC → GS arrangement should be used; when both accurate note classification and boundary detection are required, the LC & GS → BD & GS should be used.

The recognition methods investigated in this study should be applicable in all kinds of studies on animal vocalization with variable sequences that requires accurate element classification and/or element boundary detection. Essentially these methods do not depend on the particular features in acoustic data because either a DCNN and an HMM is not specialized to particular forms of inputs. Especially a DCNN is known to be good at learning good features from data without manual feature engineering [31,33], and with an HMM syntax information of variable sequences can be incorporated into recognition process.

Another achievement in this study is designing of the note & timing ER, by which recognition results of both note classes and note boundaries can be evaluated. This measure can also be used for evaluating note boundary detection without classification by setting all classes of notes to identical (ie. grouping all notes into a single class).

### Other techniques that could possibly improve the results

There are several techniques on the DCNN that could possibly decrease the validation errors. One of them is the drop-out technique, in which at each iteration of the training a certain portion of randomly chosen network nodes are turned off [39]. This procedure can be seen as training multiple networks at the same time and using average outputs of them in the recognition phase. In the current study outputs of networks trained on three different data sets in the training data were averaged in recognition. Previous studies on in image recognition have shown that averaging outputs of multiple networks with different architectures improves generalization, although it will take computational time proportional to the number of the networks [40].

In the current study we described the song syntax in Bengalese finches with a second-order Markov model in accordance with the previous study [41]. According to the previous study [42], a first-order Markov model is not sufficient for song syntax in Bengalese finches. Another study has shown that describing song syntax with a second-, third-, fourth-order Markov model resulted in qualitatively similar results [41]. The larger the order of the Markov model, the longer the computational time would be. Thus in the current study we chose second-order Markov model to describe the song syntax. There are other models that have been proposed to be suitable in describing song syntax in Bengalese finches such as an HMM [42], a simplified renewal process [43], and a k-reversed automaton [44]. Song syntax in canaries is well described by a prediction suffix tree [20]. It might result in different recognition accuracy if such syntax models are used in the global sequencing step. A k-reversible model and a prediction suffix tree can be easily implemented in our HMM framework. The simplified renewal process can be implemented as well if the number of repetition of a single note class is limited. To use a HMM as a syntax model in the global sequencing step that already uses an HMM for sequencing, a hierarchical HMM could be considered.

In the current study the 0th order discrete prolate spheroidal sequences (DPSS) was used as the taper of the short time Fourier transform to compute the sound spectrograms. Using average spectrum of the multiple tapers could generate more robust spectrograms against background noise [30,45].

There were a lot of hyper-parameters in the training procedures. Since it is virtually impossible to tune all of those parameters due to the constraint of time for training, the values of some hyper-parameters were presumably fixed in the current study. Thus, finely tuning such hyper-parameters by cross-validation within training data sets may improve the results. See the materials and methods section for the current settings of the hyper-parameters.

### Limitations and possible future directions

One obvious limitation of the present study is that pre-determined note classes and boundaries are required to train the recognizer. Although the length of songs required for training data sets is very short (~ 2 minutes), the preparation of them might be troublesome if there are tens of birds to be analyzed. To solve this problem, currently we are trying to establish the methods for unsupervised training or note clustering with features extracted by generative DCNNs such as deep generative stochastic networks [46].

Another limitation is that in the current study songs were located manually from the whole sound recordings. This is because large parts of the recordings were background noise that is not our current interest and recognizing recordings including hours of background noise takes a lot of computational time. Our methods are expected to have a capacity to locate notes in the whole recordings including long background noise, but this capacity needs to be evaluated properly in the future work. Another possible way to locate songs may be the similar method as this study assigning a single class to the whole songs and another class to the between-song silent intervals. Perhaps strict temporal resolution is not required in this song locating phase, and thus data could be down-sampled to fasten the computation.

In this study we only recognized songs in Bengalese finches. With small modifications and tunings, our methods are expected to work well in sequential vocalizations in other species because most sequential vocalizations have the three properties introduced in this paper: element classifiability, importance of timing information, and probabilistic sequencing rules. Currently we are evaluating similar recognition methods in vocalizations in other species such as other songbird species, rodents, and gibbons. However, there might be more suitable methods for songs in rodents that consist of vocal elements with long frequency-modulated sound. Also similar methods may be applied to recognizing vocalization in human babies to automatically extract both contextual information and timing information [47].

## Materials and Methods

### Ethics Statement

The experimental procedures were approved by the Institutional Animal Care and Use Committee of the University of Tokyo.

### Data acquisition

An adult male Bengalese finch (> 120 days post-hatch) was put into a sound attenuation chamber. After a habituation period of at least two days to the recording environment, sound was recorded during 14 hours of light on interval using a microphone (PRO 35, Audio-Technica Corporation, Japan), an amplifier (MicTube Duo, Alesis, United States), and an audio interface (OCTA-CAPTURE, Roland, Japan) with 32 kHz sampling rate. Light on and off intervals (14h and 10h, respectively) were controlled by an LED light. Food and water were given ad libitum.

### Data sets

Songs in 13 birds were recorded. All sequential vocalizations in the recorded sound were manually extracted by visual inspection of the sound spectrogram. Sound spectrograms were computed using short time Fourier transform with a size of 512 and a step of 32 (corresponding to 1 ms), in which frequency band between 1 and 8 kHz was used in all of the following computations. In computing spectra, the 0th order DPSS with a parameter W = 4 / 512 was used as a taper [45]. Spectrograms were mean-subtracted and divided by the standard deviation. The means and the standard deviations were computed in each training data set (defined below). In other words, this normalization was performed for each fold of the cross-validation.

Notes were located and classified manually with the help of thresholding in acoustic features and supervised machine learning such as an artificial neural network and linear discriminant analysis. All boundaries and classes that were automatically located or classified were manually corrected by visual inspection of the spectrograms. Thus, this procedure is essentially equivalent to manual annotation of all notes. The objectivity of this manual annotation was to some extent guaranteed by the low cross-validation errors shown in this paper. Non-singing calls were labeled into one class. Occasionally there were manually unclassifiable notes such as ones which do not appear to belong to any classes or which have intermediate appearance of more than one classes. Classes with notes less than 1% of the total number of notes in the songs were also labeled as unclassifiable as well.

Note sequences, separated by non-singing calls, with more than seven notes and less than 300 ms silence between notes were extracted as songs (S3 Fig). Birds with unclassifiable notes more than 1% of the total number of notes in the songs were discarded, keeping 11 birds out of 13 recorded. Then to exclude the unclassifiable notes from the data sets each song was segmented at before and after the unclassifiable notes. In spite of the definition of songs in this study stated above, segmentation at unclassifiable notes could results in shorter note sequences. Segmented songs with less than three notes were discarded. Segmented songs with more than 15 notes were further segmented so that all sequences contained less than 16 notes because uneven length of sequences would lead to inefficient computation in terms of memory management and parallelization. The segmentation of songs into note sequences with less than 16 notes could affect global sequencing with an HMM because emission probabilities of the first two notes in all sequences were assumed to follow uniform distribution (see below). In other words, recognition of the first two notes could not take advantage of the syntax information. To confirm this segmentation did not affect the recognition accuracy, we conducted global sequencing with an HMM concatenating the segmented songs in the same training or validation data sets (S3 Fig).

Note sequences were divided into three groups for three-fold cross-validation. We compared the recognition results trained on two and eight minutes of training data sets randomly selected from the sequences in non-validation set (2 / 3 of the whole data set). Note that training data sets were not continuous two or eight minutes of recorded sound, but collections of note sequences scattered across the whole recordings. Since the total sequence length differed among birds (ranging from 13.3 and 63.9 minutes), training using whole non-validation set was not performed. When a need of tuning hyper-parameters arose, the training data set was further divided into three to perform cross-validation within the training data. The hyper-parameters were set to the values that minimized the validation error in the cross-validation within the training data. Songs in each bird were individually processed because songs were largely different among birds.

### Boundary detection by amplitude and duration thresholding

In the BD → LC → GS arrangement, note onsets and offsets were detected by amplitude and duration thresholding (Fig 3). First, sound intervals with amplitude larger than a certain threshold were extracted as non-background intervals (orange bars in Fig 3). Amplitude envelope was computed as the sum of the logarithmic amplitude spectrum between the frequency band of 1 and 8 kHz in each 1 ms time bin of the spectrograms [14]. Then among the extracted non-background intervals, those with silent intervals shorter than a certain threshold between them were concatenated. Finally, intervals with duration shorter than a certain threshold were discarded. The remaining intervals were considered as the note intervals (blue bars in Fig 3). The three thresholds were determined to minimize the timing ERs among the training data set. As a result, the optimal threshold for the silent intervals was zero in all conditions in all birds, meaning that no two intervals were concatenated. Note that this threshold being zero was contingent on the data, but not the necessary condition. The optimal thresholds for sound intervals are shown in S2 Table.

Amplitude envelope can be computed in another way: full-wave rectification followed by low-pass filtering and logarithm. We compared these two methods and found that the amplitude envelope computed by sum of the logarithmic amplitude spectrum resulted in better accuracy (S2 Fig). Thus, we used this method in the following computation of the BD → LC → GS arrangement.

### Local classification with a deep convolutional network

In all three arrangements, scores for all note classes were computed for spectrograms within a fixed-length time window with a DCNN [34] (Fig 4). A DCNN serves as a feature extractor and classifier [31–33]. In the BD → LC → GS and LC → BD & GS arrangements the architecture of the network was as follows, from input to output: an input layer; three sets of convolutional layers, cascaded cross channel parametric (CCCP) pooling layers, and max-pooling layers; and two successive convolutional layers (Fig 4a and 4b). A convolutional layer is written as

$$Y_{cij}=f\left(b_{c}+\sum_{c\prime ,0\leq i\prime <h_{f},0\leq j\prime <w_{f}}W_{i\prime j\prime }X_{c\prime ,i+i\prime ,j+j\prime }\right)$$

where *X*_*cij*_ and *Y*_*cij*_ denote values in a third-order tensor, *W*_*ij*_ denotes values in a weight matrix, and *b*_*c*_ denotes values in a bias vector of the layer. The *f* denotes the activation function, *h*_*f*_ denotes the filter height, and *w*_*f*_ denotes the filter width. In a CDNN *c*, *i*, and *j* are usually called channels, rows, and columns. The CCCP pooling layers can be seen as small networks acting as activation functions in the convolutional layers [48]. They were implemented by convolutional layers with an 1×1 filter size (*h*_*f*_ = *w*_*f*_ = 1). The filter size of the first two convolutional layers and the third convolutional layer were (*h*_*f*_ = *w*_*f*_ = 5) and (*h*_*f*_ = *w*_*f*_ = 4), respectively. The filter width of the fourth convolutional layer was determined by the width of the input time window. In the BD → LC → GS and the LC → BD & GS arrangements the width of the input time window was 96 (111 ms), resulting in the filter width of the fourth convolutional layer to be *w*_*f*_ = 9. This width was determined so as to roughly cover duration of a single note. The filter height of the fourth convolutional layer was *h*_*f*_ = 11 to cover the whole input height of 112 (from 1 to 8 kHz). The filter size and the stride size of the max-pooling layers were both 2×2. The number of channels in the first three convolutional layers and the CCCP pooling layers was *c* = 16 in each. The number of channels in the fourth convolutional layer was *c* = 240. All convolutional layers and CCCP pooling layers except the last layer had rectified linear activation functions. The last convolutional layer had a softmax activation function. Thus this layer was also called a softmax layer. In the LC & GS → BD & GS arrangement, another convolutional layer was inserted before the last layer, hoping to capture the syntax information that spanned over more than one note (Fig 4c). The filter width of the inserted layer was *w*_*f*_ = 25, corresponding to the input window width of 288 (303 ms), roughly covering the duration of three successive notes. The aim of inserting this layer was to integrate the local single-note information in the fourth convolutional layer into the global syntax information over three notes. In other words, it tries to implicitly combine the outputs of local classification and the global syntax in the form of a trigram syntax model. To ensure proper classification under the fourth convolutional layer, the network without the inserted layer was trained before inserting additional fifth convolutional layer and training the whole network.

Updating of parameters (weights and biases) was performed by a simple stochastic gradient descent method with a cross-entropy cost function. The learning rate was determined in each training data set as follows. First, the initial search of the learning rate was conducted using 2 / 3 of the training data set for training and the other 1 / 3 for validation. Training was conducted with various learning rates from 0.001 to 0.04. The initial learning rate was set to the value that achieved the lowest validation error in one of the first 32 training iterations. Then the full training was conducted on the 2 / 3 of the training data as long as the validation error in the other 1 / 3 kept decreasing. When the validation error stopped decreasing, the learning rate was decreased by half and the training was continued. This procedure was repeated three times. The training was performed on the three different combinations of the sub-training and sub-validation data, yielding three parameter sets in each training data set. Parameter update was performed iteratively for small data set (called mini-batch) selected randomly from the training data. Data in each mini-batch was selected so that total length in each mini-batch did not exceed 32 s. In the recognition phase, the outputs of those three networks were averaged.

The network weights and biases were initialized according to [49]. The initial weights were sampled from a Gaussian distribution whose mean was zero and standard deviation was the square root of two divided by the number of incoming connections to the particular node. All biases were initialized to zero. The random seed for the initial weights were searched at the same time with the initial search of the learning rate.

In the case of BD → LC → GS arrangement, the size of the softmax layer was the number of note classes. In the other two arrangements, to perform the following boundary detection with an HMM, the size of the softmax layer was three times the number of note classes plus one corresponding to silence.

### Boundary detection and global sequencing with a hidden Markov model

In the BD → LC → GS arrangement, the outputs of local classification were combined with the global syntax information with an HMM [25,37] (Fig 1a). Generally when an HMM are combined with a DCNN, the outputs of the DCNN are considered as the posterior probabilities of the hidden states [25,50]. In the current study, song syntax was described with a second-order Markov model [41] (Fig 5a). To describe the syntax of note sequences with a second-order Markov model, the structure of an HMM was modified so that the transition between hidden states were mediated by generation of a symbol corresponding to the note class, and that the outputs of the DCNN were considered as the posterior probabilities of the symbols.

$$\begin{array}{l} \text{YZ }\sim \;P(\text{Z }|\text{ XY}),\\ DCNN\;output=P(Z\;|\;spectrogram), \end{array}$$

where X, Y, and Z denote note classes.

The transition probability between hidden states was computed from the training data set with smoothing by adding a constant value before dividing by the sum.

$$P(\text{Z}|\text{XY})=\frac{P_{0}(\text{Z}|\text{XY})+\alpha }{\sum_{\text{Z'}}\left(P_{0}(\text{Z'}|\text{XY})+\alpha \right)}$$

where *P*(Z|XY) and *P*_*0*_(Z|XY) denote smoothed and original transition probability of the note class Z after notes X and Y, respectively. The *α* is a smoothing constant, which was determined by cross-validation within the training data set. The transition probabilities from the initial state and the next state of the initial state were assumed to be uniformly distributed. The outputs of the DCNN were averaged over each sound interval detected by thresholding to obtain the posterior probability of the hidden states in each interval. The optimal state sequences for the computed posterior probabilities were estimated by Viterbi algorithm, which were then converted into the label sequences of the note classes.

In the CL → BD & GS and the CL & GS → BD & GS arrangements, boundary detection was performed simultaneously with global sequencing (Fig 1b and 1c, and S4 Fig). To accurately detect note boundaries, each note was divided into three parts with the same duration (Fig 8), and each state was divided into four: first three of which emitted three parts of a note and last of which emitted the silent interval between notes. These divided sub-states are connected in the left-to-right manner including self-transitions (Fig 5b). The last two sub-states had the connections to the next state, which correspond to the transition from one note to another. The transition probabilities in these transitions followed the second-order note transition probabilities in the training data set with smoothing. The other transition probabilities (transition probabilities from the initial state, those from the next state of the initial state, and transition probabilities from a sub-state to the same sub-state or a next sub-state) were assumed to be uniformly distributed. Specifically, transition probabilities from a state XY_i_ (i = 1 or 2) were

P(XYi|XYi) = P(XYi+1|XYi) = 0.5

where XY_i_ denotes an i-th sub-state. Transition probabilities from a state XY_3_ were as follows:

$$P({\text{XY}}_{\text{3}}|{\text{XY}}_{\text{3}})=P({\text{XY}}_{\text{s}}|{\text{XY}}_{\text{3}})=\frac{1}{n+2}$$

$$P({\text{YZ}}_{\text{1}}|{\text{XY}}_{\text{3}})=\frac{n}{n+2}P(\text{Z}|\text{XY})$$

where XY_3_ and XY_s_ denotes third and fourth sub-states, and *n* denotes the number of note classes. Transition probabilities from a state XY_s_ were as follows:

$$P({\text{XY}}_{\text{s}}|{\text{XY}}_{\text{s}})=\frac{1}{n+1}$$

$$P({\text{YZ}}_{\text{1}}|{\text{XY}}_{\text{s}})=\frac{n}{n+1}P(\text{Z}|\text{XY})$$

Dividing each note into more than two parts was crucial. This is because if no division was made in a note, the HMM would not be able to distinguish two notes with a very small silent interval from one long note by mistakenly inserting or skipping short silences (notes A and C in Fig 8b). If notes were divided into two parts, small misclassifications of two sub-divisions would cause unwanted onsets and offsets at the positions of the misclassifications. (notes B in Fig 8c) because in the HMM transitions from the second sub-states to the first sub-states were allowed. In the case of notes B in Fig 8c, unwanted note boundary was recognized at the transition from the state AB_2_ to the state BB_1_. If notes were divided into three, transitions from the second sub-states to the first sub-states were not allowed by the left-to-right constraints. Thus, in the case of note B in Fig 8d, the optimal state sequences were from AB_2_ to AB_3_, not to BB_1_, even if the outputs of the DCNN at the time were large for the first sub-division in note B.

In general cases of recognition in an HMM with a DCNN, the outputs of the DCNN are considered as the posterior probability of the hidden states given the acoustic data, *P*(Z|*spectrogram*). The posterior probability is often converted into the emission probability of the acoustic data given the hidden states by Bayes' rule [25,50]:

$$P(spectrogram|\text{Z})\propto \frac{P(\text{Z}|spectrogram)}{P(\text{Z})}$$

In this study we chose whether to conduct this conversion according to the cross-validation within the training data sets. When the outputs of the DCNN was not converted into the emission probability, the emission probability was assumed to equal to *P*(Z|*spectrogram*).

### Computation

All computations and sound recording were implemented in the custom written java and cuda program. The source code is available at https://github.com/takuya-koumura/birdsong-recognition. Training of the DCNN and recognition were conducted using cuDCNN library on graphic processors (GTX 970 or 980, NVIDIA, United States).

## Supporting Information

S1 Fig Identification errors and duration errors. In the current study, we computed three types of validation errors: note error rates (ERs), timing ERs, and timing & note ERs. Although these three error rates are well-defined and sound measures to capture the corresponding accuracies, these errors are not very much straightforward or intuitive. Thus, we also computed other measures called identification ERs, duration ERs of notes, and duration ERs of silent gaps.
Identification ERs was defined by the fraction of incorrectly recognized notes. The incorrectly recognized notes were defined by the notes in the true sequences with the nearest notes in the output sequences to which incorrect classes were assigned, or by the notes in the true sequences that had two notes in equal distance in the output sequences.
Duration ERs of notes were defined by sum of the duration differences between correctly recognized notes and those of the nearest notes in the output sequences, divided by the total length of the correctly recognized notes. The correctly recognized notes were defined by the remainders of the incorrectly recognized notes defined above.
Duration ERs of silent gaps were defined as sum of the duration differences between two silent gaps in the true sequences and the output sequences that were nearest to each other, divided by the total length of silent gaps in the true sequences.
Confusion matrices and duration ERs for each note class in each bird were shown in S1 Table.
(a) Identification ERs in each arrangements with two and eight minutes of training data. ERs in each bird are shown in open circles. (b) Duration ERs of notes. (c) Duration ERs of silent gaps.
(EPS)

S2 Fig Comparison of the two methods for computing amplitude envelope. In the BD → LC → GS arrangement note boundaries were detected using amplitude envelope. Amplitude envelope was computed as the sum of the logarithmic amplitude spectrum between the frequency band of 1 and 8 kHz in each 1 ms time bin of the spectrograms. However, amplitude envelope can be computed in another way: full-wave rectification followed by low-pass filtering and logarithm. Here we compared the accuracy of boundary detection by these two methods. The cut-off frequency of the low-pass filter was 200Hz.
Timing ERs were larger in the methods using low-pass filter in all birds. Thus in the current study we used the method using spectrogram in the BD → LC → GS arrangement. ERs in each bird are shown in open circles. ***: *p* < 0.001.
(EPS)

S3 Fig Song definition and segmentation. (a) Schematic representation of the procedure to extract note sequences. Gray, white, and black rectangles represents song notes, unclassifiable notes, and non-singing calls. Horizontal bars represent extracted or segmented note sequences. (b) Comparison of the validation error rates computed with or without song segmentation. ERs in each bird are shown in open circles. ns.: not significant.
(EPS)

S4 Fig Notes and HMM states dividing. An example of computation procedure of the global sequencing step. From top to bottom: input spectrogram, local classification outputs, HMM states, and output sequences. Rows in the classification outputs represent three note classes (A, B, and C) and silence (S). Selected HMM states by Viterbi algorithm were indicated by black rectangles. The letter “e” in the HMM states denotes the initial states. Notes in output sequences were represented by gray rectangles.
(EPS)

S1 Table Confusion matrices and duration errors. (PDF)

S2 Table Optimal thresholds for sound intervals in boundary detection. (PDF)
