# Supplementary material for: Automatic Recognition of Element Classes and Boundaries in the Birdsong with Variable Sequences
Source: PLoS One. 2016 Jul 21;11(7):e0159188. doi: 10.1371/journal.pone.0159188 (PMC4956110; doi:10.1371/journal.pone.0159188)
Supplement: S1 Table — (PDF) [file pone.0159188.s005.pdf]

Bird 0

Ground truth \ Answer	A	B	C	D	E	F	G	H	I	Duration
A	99	< 1								2.6
B	< 1	98							< 1	2.4
C		< 1	99							1.4
D	1			98						2.2
E					100					2.7
F						99	< 1			1.6
G		< 1				< 1	99			1.3
H	< 1			< 1				98	< 1	3.5
I	< 1	< 1						< 1	98	3.0

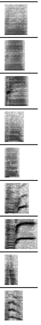

Bird 1

Ground truth \ Answer	A	B	C	D	E	F	G	H	I	J	K	L	Duration
A	99		< 1	< 1						< 1	< 1	< 1	3.8
B	< 1	99											4.9
C	< 1		99		< 1								2.4
D	< 1			99									2.0
E			< 1		99	< 1							2.3
F	< 1	< 1	< 1			99							3.4
G		< 1					99						3.7
H								99				< 1	3.3
I									100				1.2
J	< 1									99	< 1		2.7
K										< 1	99		2.2
L	< 1							< 1		< 1		98	3.7

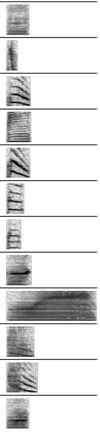

Bird 2

Ground truth \ Answer	A	B	C	D	E	F	G	H	I	J	K	L	M	N	O	P	Q	Duration
A	99	< 1	< 1														< 1	2.8
B	1	98																1.8
C	3		96															3.4
D		< 1		99														1.9
E			< 1		99													1.9
F						100												1.4
G							100											1.5
H								100										2.3
I									100									1.9
J										100								1.8
K			< 1								99							1.8
L												100						2.3
M													100					2.3
N														100				1.6
O															100			1.1
P																100		2.2
Q																	100	2.2

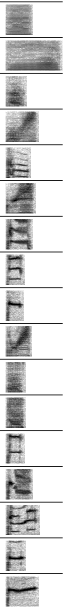

Bird 3

Ground truth \ Answer	A	B	C	D	E	F	G	H	I	Duration
A	99	< 1		< 1	< 1					2.3
B	< 1	95		4						5.8
C	< 1	< 1	99							1.6
D		< 1		99						5.3
E	< 1				99					1.8
F						100				2.7
G							100			6.3
H								100		3.9
I	< 1								99	3.0

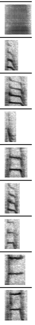

Bird 4

Ground truth \ Answer	A	B	C	D	E	Duration
A	99	< 1			< 1	3.6
B	< 1	99			< 1	3.3
C	< 1		99		< 1	1.3
D	< 1		< 1	99	< 1	1.4
E	3				96	3.5

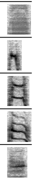

S1 Table | Confusion matrices and duration errors.

Bird 5

Ground truth \ Answer	A	B	C	D	E	F	G	Duration
A	99	< 1				< 1		2.6
B	< 1	99		< 1				2.4
C			100					1.9
D		< 1		99				1.8
E				< 1	99			1.2
F						100		1.0
G							100	2.7

Bird 6

Ground truth \ Answer	A	B	C	D	E	Duration
A	99			< 1		2.7
B	< 1	99				1.1
C	< 1	< 1	99	< 1		1.4
D	< 1		< 1	99		2.7
E					100	3.8

Bird 7

Ground truth \ Answer	A	B	C	D	E	F	G	Duration
A	100							2.7
B	< 1	99	< 1					2.2
C	< 1	< 1	99					1.3
D				100				1.8
E					100			1.6
F	< 1					99		1.4
G	1						98	4.7

Bird 8

Ground truth \ Answer	A	B	C	D	Duration
A	100				3.1
B		100			1.0
C	1		98		1.7
D				100	1.3

Bird 9

Ground truth \ Answer	A	B	C	D	E	F	Duration
A	99	< 1			< 1	< 1	5.5
B	< 1	99					4.3
C			100				1.9
D	< 1	< 1		99			2.8
E					100		0.9
F						100	1.2

Bird 10

Ground truth \ Answer	A	B	C	D	E	F	G	H	I	J	K	L	Duration
A	98	1										< 1	4.2
B	< 1	98										1	2.2
C	< 1		99							< 1			1.4
D				100									1.7
E					100								1.4
F						100							1.9
G							100						2.2
H	< 1							99					1.4
I									100				1.5
J										100			0.8
K											100		1.0
L	1	1										96	1.8

S1 Table (continued).

Note: Confusion matrix and duration error for each bird. Each row indicates the true class. Each column indicates the output class, the duration ER, and the representative spectrogram of the note. The values are percentages. The values less than 1% are shown as < 1. Zero percent is not shown.
