# Supplementary material for: Automatic Recognition of Element Classes and Boundaries in the Birdsong with Variable Sequences
Source: PLoS One. 2016 Jul 21;11(7):e0159188. doi: 10.1371/journal.pone.0159188 (PMC4956110; doi:10.1371/journal.pone.0159188)
Supplement: S2 Table — (PDF) [file pone.0159188.s006.pdf]

	Training data length (minutes)	
Bird ID	2	8
Bird 0	32.0, 33.0, 33.0	32.0, 29.0, 30.0
Bird 1	35.0, 32.0, 31.0	30.0, 32.0, 32.0
Bird 2	35.0, 32.0, 32.0	31.0, 32.0, 36.0
Bird 3	32.0, 30.0, 30.0	29.0, 31.0, 29.0
Bird 4	32.0, 32.0, 42.0	37.0, 32.0, 41.0
Bird 5	25.0, 26.0, 35.0	24.0, 26.0, 26.0
Bird 6	41.0, 43.0, 42.0	41.0, 41.0, 41.0
Bird 7	36.0, 31.0, 35.0	34.0, 30.0, 30.0
Bird 8	37.0, 35.0, 36.0	36.0, 35.0, 36.0
Bird 9	40.0, 40.0, 36.0	34.0, 36.0, 34.0

S2 Table | Optimal thresholds for sound intervals in boundary detection.

Note: Three values for three-fold cross validation are given in each training data length. The values are in milli-seconds.
